# Combining Coarse-Grained Simulations and Single Molecule Analysis Reveals a Three-State Folding Model of the Guanidine-II Riboswitch

**DOI:** 10.3389/fmolb.2022.826505

**Published:** 2022-04-19

**Authors:** Christin Fuks, Sebastian Falkner, Nadine Schwierz, Martin Hengesbach

**Affiliations:** ^1^ Institute for Organic Chemistry and Chemical Biology, Goethe-University Frankfurt, Frankfurt am Main, Germany; ^2^ Department of Theoretical Biophysics, Max Planck Institute of Biophysics, Frankfurt am Main, Germany; ^3^ Computational and Soft Matter Physics, University of Vienna, Vienna, VIA, Austria

**Keywords:** single-molecule FRET, coarse-grained simulations, riboswitch, guanidine-II riboswitch, RNA dynamics

## Abstract

Riboswitch RNAs regulate gene expression by conformational changes induced by environmental conditions and specific ligand binding. The guanidine-II riboswitch is proposed to bind the small molecule guanidinium and to subsequently form a kissing loop interaction between the P1 and P2 hairpins. While an interaction was shown for isolated hairpins in crystallization and electron paramagnetic resonance experiments, an intrastrand kissing loop formation has not been demonstrated. Here, we report the first evidence of this interaction *in cis* in a ligand and Mg^2+^ dependent manner. Using single-molecule FRET spectroscopy and detailed structural information from coarse-grained simulations, we observe and characterize three interconvertible states representing an open and kissing loop conformation as well as a novel Mg^2+^ dependent state for the guanidine-II riboswitch from *E. coli*. The results further substantiate the proposed switching mechanism and provide detailed insight into the regulation mechanism for the guanidine-II riboswitch class. Combining single molecule experiments and coarse-grained simulations therefore provides a promising perspective in resolving the conformational changes induced by environmental conditions and to yield molecular insights into RNA regulation.

## Introduction

Riboswitches are *cis*-regulatory elements that are located in the 5′ untranslated region (5′ UTR) of bacterial mRNA, affecting the expression of the downstream gene. They are generally comprised of an aptamer domain and an expression platform. The aptamer domain is responsible for specific ligand binding, whereas part of the expression platform can form distinct structures that trigger the genetic decision. By structurally coupling the aptamer domain with the expression platform, riboswitches are able to execute their regulatory function. There is a wide spectrum of metabolites that can be bound by the respective aptamer domain, ranging from ions (Baker et al., 2012; Sherlock and Breaker 2017), amino acids (Sudarsan et al., 2003), nucleotides (Mandal and Breaker 2004) and cofactors (Winkler et al., 2002) to large biomolecules such as tRNAs (Green et al., 2010). In general, two functional types of riboswitches can be discriminated: the transcriptional and translational riboswitches. In transcriptional riboswitches the expression platform contains a terminator sequence that halts transcription upon correct folding. In contrast, translational riboswitches contain an anti-Shine-Dalgarno (SD) sequence (Winkler and Breaker 2003). Sequestering of the SD sequence prevents ribosome binding and thus translation initiation. In both cases ligand binding to the aptamer could either switch gene expression on or off, based on the type of riboswitch. This positive or negative feedback loop allows utilization of specific biosynthetic pathways [such as in the 2′dG riboswitch (Kim et al., 2007)], or elimination of toxic substances [such as the fluoride riboswitch (Baker et al., 2012)].

**FIGURE 1:**
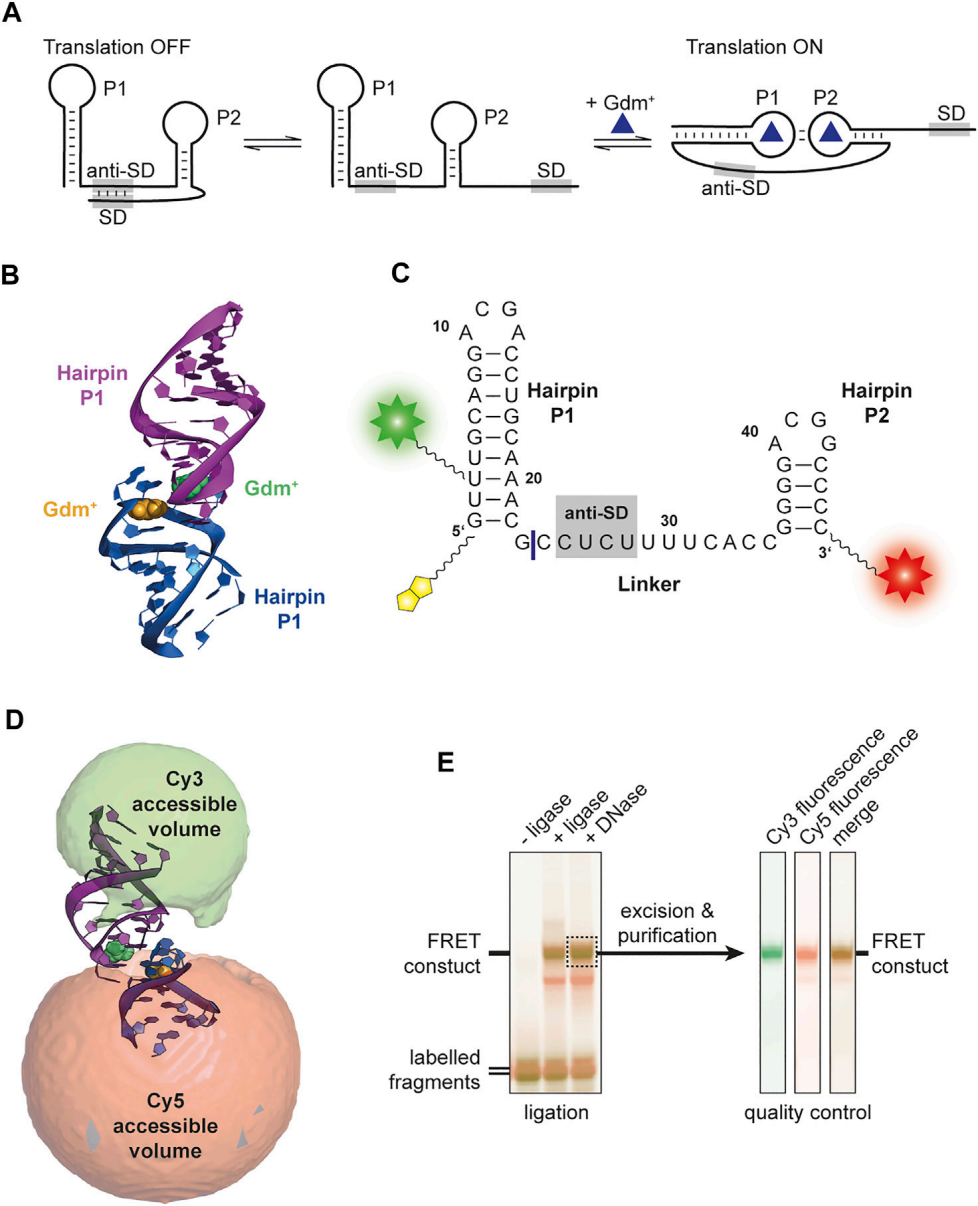
**(A)** Proposed translational regulation model. Gdm^+^ binding leads to a kissing loop interaction and sequestering of the anti-Shine-Dalgarno sequence thus allowing translation of adjacent genes. **(B)** Crystal structure of *E. coli* SugE guanidine-II riboswitch P1 homodimer (PDB ID: 5NDI) (Huang et al., 2017). The individual P1 RNA molecules are shown in blue and purple, respectively and the Gdm^+^ ligands bound to each loop region are depicted in yellow and green. **(C)** Sequence of the FRET construct used in this work. The biotin moiety is illustrated in yellow and the Cy3 and Cy5 attachments sites are shown as a green and red star, respectively. The ligation site between the residues 23 and 24 is shown as a blue line. **(D)** Accessible volume of the Cy 3 (green) and Cy5 (red) fluorophores calculated with FPS. One stem from the crystal structure was shortened according to the four base pairs in P2. **(E)** Denaturing PAGE analysis of the ligation reaction of the fluorophore labelled 23mer and 24mer fragments to yield the FRET construct (left). The band containing the successfully ligated full length 47mer aptamer with both fluorophores attached (dashed box) was excised and purified. Additional denaturing PAGE analysis of the dual fluorescently labelled, purified construct used for smFRET exeriments was performed as quality control (right).

So far, four classes of riboswitches have been identified that bind the cationic molecule guanidinium (Gdm^+^): guanidine-I (Breaker et al., 2017), -II (Sherlock et al., 2017), -III (Sherlock and Breaker 2017) and -IV (Salvail et al., 2020). The corresponding genes are in most cases involved in Gdm^+^ detoxification, and code for proteins like guanidine carboxylases or multidrug efflux pumps (e. g., SugE). While guanidine-I and -IV are transcriptionally regulated riboswitches, guanidine-II and -III are proposed to be translational riboswitches. The latter having an additional level of regulation via protection from RNase E degradation (Richards and Belasco 2021). The guanidine-II riboswitch is the shortest representative of guanidine riboswitches classes, and was named mini-*ykkC* prior to Gdm^+^ being identified as the ligand in 2017 (Sherlock et al., 2017). It consists of two GC-rich hairpins termed P1 and P2, both containing a conserved ACGR loop motif. It has been proposed that this class features a translational regulation mechanism because the two hairpins are connected with a linker containing a putative anti-SD sequence (Reiss and Strobel 2017). So far, in-line probing experiments (Sherlock et al., 2017), crystallization (Huang et al., 2017; Reiss and Strobel 2017; Huang et al., 2019) and molecular dynamics (Steuer et al., 2021) analyses have suggested a kissing loop formation through canonical CG base pairs upon binding of the ligand Gdm^+^ to the loop region (Figures 1A,B) associated with a rearrangement of those nucleotides compared to the unbound-like state. This interaction supposedly sequesters the anti-SD sequence, exposing the SD sequence and thus facilitating translation of the downstream gene (Figure 1A). Mg^2+^ binding sites have been identified in each of the two stems (Huang et al., 2017; Schamber et al., 2021). Crystallization could however only show homo-dimerization of isolated hairpins. Using electron paramagnetic resonance (EPR) it was shown that RNAs comprising either hairpins P1 or P2 could form homo-as well as heterodimers (Wuebben et al., 2020). Recently, a study combining nuclear magnetic resonance (NMR) and small angle X-ray scattering (SAXS) showed interstrand kissing loop formation in an *in vitro* environment (Schamber et al., 2021).

Until now, there was however no direct evidence that an interaction *in cis* between the two hairpins can form in a functional RNA (Wuebben et al., 2020) and that this interaction is regulated by ligand binding. To fill this gap, we combined single-molecule Förster resonance energy transfer (smFRET) spectroscopy and coarse-grained simulations. Our work allows us to also discuss the limitations and mutual benefits of these two complementary approaches. The results show that the riboswitch aptamer domain can adopt three different conformational states, including a ligand dependent state that involves intrastrand kissing loop formation.

## Materials and Methods

### Construct Design, Dye Attachment, and FRET Positioning and Screening Software

For this work the wild-type sequence of the *E. coli* SugE guanidine-II riboswitch aptamer was used (Figure 1C). The construct contained the P1 and P2 hairpin as well as the native linker connecting both hairpins. The 5′ C1 was exchanged with a G to stabilize the hairpin. Labelling sites were chosen at U3 via a C5 amino-allyl modification and the 3′ phosphate with a C6 amino-modifier. For immobilization, a biotin modifier was used at the 5′ end. The sequence was split into a 23mer and 24mer to allow separate labelling.

For FRET efficiency prediction, the FRET positioning and screening software (FPS) (Kalinin et al., 2012) was used. The crystal structure of the *E. coli* P1 hairpin homodimer [PDB: 5NDI (Huang et al., 2017)] was used as a template, and the FPS-derived FRET efficiency was used only for identification of the kissing-loop conformation accordingly. Since the native stems deviate in their length from this structure, we corrected one helix of this structure for the purpose of the FPS simulations to four nucleotides in order to correctly simulate the native riboswitch RNA hairpins. As parameters for the FPS simulation, we used previously established values (Schmidt et al., 2018): For Cy3, dye radii of 6.8, 3.0, and 1.5 Å, respectively, were used. Cy5 was calculated with 11.0, 3.0, and 1.5 Å, respectively. The 12-atom flexible linkers were described with 22.3 and 4.5 Å for 5′ modification (C5 of amino-allyl uridine), and 27.1 and 4.5 Å for the 3′ modification (starting from the 3′ oxygen). The Förster radius for the Cy3/Cy5 pair was set to 60 Å (Murphy et al., 2004). The program was used to calculate the accessible volume clouds as depicted in Figure 1D, as well as the expected FRET efficiencies and average distances between the dyes.

### RNA Synthesis: Labelling

Modified RNAs for the FRET construct were purchased in two fragments (Dharmacon) (5′ fragment: Biotin-GU(5-NH_2_-U) UGC AGG ACG ACC UGC AAA CG, 3′ fragment: P-CCU CUU UUC ACC GGG GAC GGC CCC-C_6_NH_2_). 30 nmol of each RNA were ethanol precipitated, and subsequently resuspended in 20 µl freshly prepared 0.1 M NaHCO_3_ (pH 8.0). Cy3 or Cy5 amine-reactive dyes (Amersham CyDye Mono-Reactive Dye Packs, GE Healthcare) were dissolved in 20 µl DMSO. Labelling was achieved by mixing the two solutions and incubation of the RNA with the respective dye for 90 min (3′ fragment) or 3 h (5′ fragment) at room temperature under light protection. RNA was precipitated and dissolved in 300 µl deprotection buffer (100 mM AcOH adjusted to pH 3.8 with TEMED) and incubated at 60°C for 40 min (3′ fragment) or 2 h (5′ fragment). Deprotected RNA was precipitated, and non-biotinylated RNA was dissolved in 0.1 M TEAA (pH 7.0) and purified via reverse phase chromatography with an Äkta Basic system using a C8 column (Kromasil 100 C8 7 µm 250 × 4.6 mm). A gradient from 100% TEAA buffer to 50% MeCN was applied. Fractions with labelled RNA were collected and precipitated.

### RNA Synthesis: Ligation and Purification

The FRET construct was synthesized through splinted ligation of the two fluorophore labelled fragments. All nucleic acid components had a final concentration of 10 µM each. RNA fragments were dissolved in water, heated to 95°C for 2 min and placed on ice. The DNA splint (TGG GGC CGT CCC CGG TGA AAA GAG GCG TTT GCA GGT CGT CCT GCA AAC CTA TAG TGA GTC GTA TTA) and T4 ligase buffer (50 mM TRIS/Cl, 10 mM MgCl_2_, 1 mM ATP, 10 mM DTT, pH 7.5) were added and the mixture incubated at 85°C for 3 min. After slowly cooling down T4 DNA ligase (final concentration of 40 U/mL, NEB) was added and the reaction was performed for 2 h at room temperature. 1 U Turbo DNase (Invitrogen) was added and incubated for 30 min at 37°C, and subsequently extracted using phenol/ether extraction and precipitated. The FRET construct containing the desired sequence was separated via denaturing PAGE, eluted and ethanol precipitated.

### smFRET Measurements

Microscope slides (quartz) and cover slips were cleaned with nitrogen plasma for 10 min. Channels were made by aligning parafilm stripes on the slide, covering it with the coverslip and heating everything to 80°C for 30 s. Cooled down channels were filled with 1 mg/ml biotin labelled bovine serum albumin (BSA, Sigma-Aldrich) in T50 buffer (10 mM Tris/Cl, 50 mM NaCl, pH 8.0) and incubated for 2 min. Channels were then washed with 50 µl of T50 before incubation with 0.2 mg/ml streptavidin in T50 for 2 min. Channels were washed with 50 mM Tris/Cl (pH 7.4) with the respective Mg^2+^ and Gdm^+^ concentration. 100 pM RNA was folded in the respective buffer by incubation at 95°C for 2 min and cooling on ice for 5 min. RNA was flushed into the channel for immobilization. Prior to the measurement, the channel was rinsed with imaging buffer [sample conditions in Tris buffer, 10% (w/v) D-(+)-glucose, 80 μg/ml glucose oxidase, 20 μg/ml catalase, and Trolox (saturated)].

An objective-type spinning-spot total internal reflection microscopy setup with an EMCCD camera (iXon, Andor Technology) with 532 nm laser (green laser) excitation with an integration time of 100 ms at 22°C was used for smFRET measurements. This ensures that molecules lacking a Cy3 modification do not contribute to the data collected in these experiments. For histograms 20 frames with green excitation were recorded. For kinetic data and verification of single step photobleaching movies of up to 7 min were recorded.

In order to improve the data basis for our analysis, we used a larger number of short movies (2 s) to calculate FRET efficiencies for histograms. FRET efficiencies were binned to a bin size of 0.05. The donor only peak was fitted with a Gaussian fit, and subtracted from the data. The remaining data was plotted into histograms. For these histograms, three states were fitted with a Gaussian fit using OriginPro 2018b (Northampton). The fractions of the individual states were calculated from the ratios of the area under the individual curves. For kinetic data, traces before photobleaching were manually selected for consistency, anticorrelated dye behaviour, and single-step photobleaching. The selected traces were stitched to a single trace of 50,000 datapoints. Since the number of transitions for each molecule was significantly higher than the number of stitched molecules, this only marginally affects the kinetic information extracted from these stitched traces. In cases where this prerequisite was not fulfilled, we indicated “not determined” (n.d.). Hidden Markov modelling software (HaMMy) (McKinney et al., 2006) was then applied using a three state model. Fitting the traces with a five state model did not result in additional, discernible FRET states (data not shown). Dwell times for each transition were fitted with an exponential decay and rate constants (k) were calculated using OriginPro 2018b.

### Coarse-Grained Simulations

The simulation construct was modeled based on a crystal structure of the *E. coli* guanidine-II riboswitch P1 stem-loop dimer (PDB: 5NDI) (Huang et al., 2017). One loop was shortened to the length of the wild type P2 stem-loop. Base pairs were mutated to match the FRET construct using Chimera (Pettersen et al., 2004). The linker between the P1 and P2 stem-loops was modeled using ModeRNA (Rother et al., 2011). The coarse-grained RNA simulations were performed using a three-interaction site model (TIS) developed by Thirumalai and coworkers (Hyeon and Thirumalai 2005; Denesyuk and Thirumalai 2013, 2015; Hori et al., 2016; Nguyen et al., 2019). For our present study, TIS is particularly suited since it is computational efficient allowing us the investigation of folding/unfolding transitions while reproducing the folding thermodynamics with good accuracy (Denesyuk and Thirumalai 2013). In the TIS model, the intramolecular attractive interactions are defined based on the residues that appear in the native structure. This description, inherent to all Gō-like models, ensures that the native structure is the minimum energy structure (Abe and Go, 1981). Native hydrogen bonds and tertiary stacks in the simulation construct were defined based on the crystal structure of the P1 stem-loop dimer (Huang et al., 2017). In order to capture folding intermediates that are not stabilized by native interactions, non-native secondary structure interactions were included via the base-stacking interactions of consecutive nucleotides and hydrogen-bond interactions between all nucleobases (Denesyuk and Thirumalai 2015). A non-interacting adenine was added capping the structure at the 3′-end. The RNA was placed in a cubic box with an edge length of 70 nm. Numerical integration of the equations of motion was performed using the leap-frog algorithm with time step h = 0.05 τ where τ = 50 fs at 298.15 K as in previous work with TIS (Hyeon and Thirumalai 2005). The simulations were performed in the low friction regime dynamics to increase the sampling efficiency of the conformations, in which the viscosity of water was reduced 100 times (Honeycutt and Thirumalai 1992). The cut-off for electrostatic interactions was set to 3 nm. Magnesium was included explicitly via the effective Magnesium-phosphate interaction potential derived from RISM theory (Nguyen et al., 2019) **.** Simulations were performed at six different concentrations ranging from 0 to 10 mM Mg^2+^. Each concentration was simulated for 1.5 × 10^9^ steps corresponding to 3.75 μs. In total 22.5 μs of simulation time was used. Monovalent ions were included implicitly at a concentration of 50 mM. To compare the simulations to experiments, the FRET efficiency was calculated. Structures from the simulations were backmapped to an atomistic representation. Accessible volumes of the FRET dyes and corresponding mean FRET efficiencies were estimated using *avtraj* (Dimura et al., 2016). Dye parameters were matched to the previously stated FPS parameters. The resulting FRET efficiencies were subsequently binned using the same bin size (0.05) as in the experiments and fitted with the same routine described above. For comparison with experimental data the most probable FRET efficiency of each state is obtained from the maximum of the fitted Gaussian.

### Secondary Structure Prediction

For secondary structure predictions, the online folding tools Mfold RNA folding Form version 2.3 (Zuker 2003) and Vienna RNAfold (Lorenz et al., 2011) were used. Structure predictions were performed for both the 47mer RNA sequence with a stabilizing G1 used in this work as well as the native aptamer.

## Results

### Construct Design and Synthesis

Several crystal structures have shown the formation of a kissing loop interaction involving canonical C-G base pairs between isolated hairpins (Huang et al., 2017; Reiss and Strobel 2017; Huang et al., 2019), resulting in homodimeric structures. However, two problems can be envisioned when transferring these findings to functional constructs. First, the two stems in all natural riboswitches have different sequences (Weinberg et al., 2007). Secondly, the two hairpins are linked with a presumably regulatory anti-Shine-Dalgarno sequence. It is therefore unclear whether the interactions found in the crystal structures can actually be transferred to the natural riboswitch. Therefore, a linked construct comprised of the native hairpins is required to resolve whether the interaction between P1 and P2 helices can occur *in cis*.

Based on the available structural information from crystal structures (Huang et al., 2017) as well as in-line probing experiments (Sherlock et al., 2017), an RNA FRET construct was designed. For this, a 47mer RNA sequence of the guanidine-II riboswitch complete aptamer region upstream of the *E. coli sugE* gene was selected (Figure 1C). Fluorophore attachment sites were placed in helical regions of both hairpins expecting them to assume a defined distance upon kissing loop formation, supposedly creating a signature FRET efficiency. A native U of the P1 stem was modified at position C5, and the second modification was introduced at the 3′ phosphate.

In order to identify the expected FRET efficiency of this construct in a potential kissing loop conformation (such as the one in the crystal structure), we modelled the available conformational states for the dyes with the FPS software package. This revealed a FRET efficiency of 0.72 (Figure 1D). Based on these results the RNA fragments were designed, and the construct successfully synthesized. We therefore divided the natural aptamer domain into two fragments corresponding to the 23mer P1 hairpin and the 24mer linker and P2 region (Figure 1C). The 5′ fragment had an additional conjugated biotin to allow immobilization required for smFRET measurements. Fragments were successfully labelled with Cy3 and Cy5 using NHS-chemistry, respectively. The final FRET construct was synthesized via subsequent splinted ligation of the dye-labelled fragments and denaturing PAGE purification of the double labelled RNA (Figure 1E).

### Initial smFRET Characterization

**FIGURE 2:**
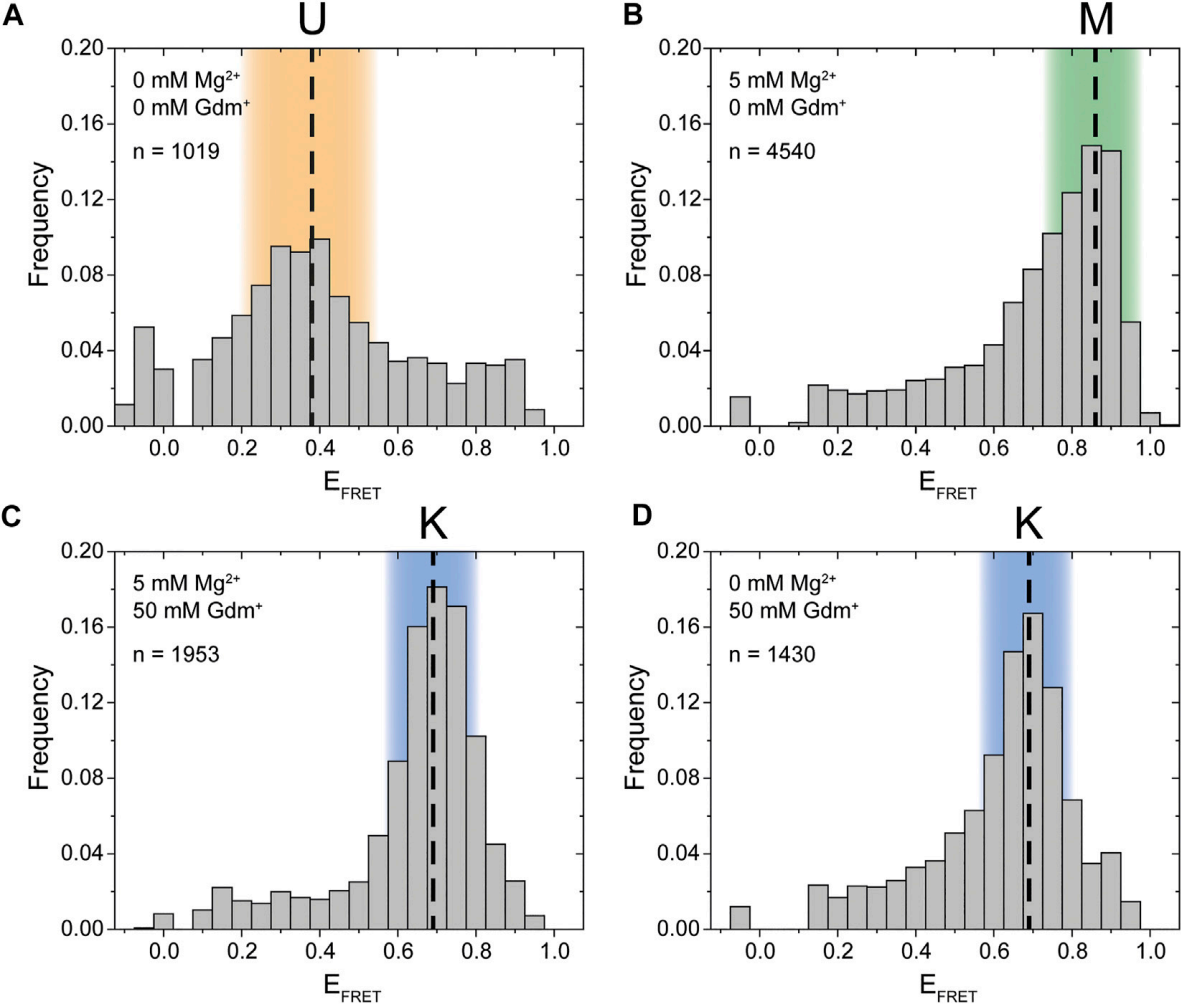
Initial smFRET experiments. The histograms show the populations at different FRET efficiencies. **(A)** in the absence of ions **(B)** with only Mg^2+^ present **(C)** with both Mg^2+^ and ligand present and **(D)** with only the ligand present. The U-state is highlighted in orange, the K-state in blue and the M-state in green.

Synthesis of this FRET construct now enables investigation whether P1 and P2 interact *in cis* using smFRET. Initially, we tested the FRET efficiencies and their response to selected Mg^2+^ ion and Gdm^+^ ligand conditions. In the absence of both ligand and Mg^2+^ the majority of molecules resided in a low FRET state (E_FRET_ ≈ 0.38) (Figure 2A). This low FRET state corresponds to a distance between the dyes that is larger than what would be expected for the conformation modelled from the crystal structure. As shown in the following, this state corresponds to an unfolded conformation, which we term the U-state.

In presence of 5 mM Mg^2+^, the riboswitch folds into a high FRET state (E_FRET_ ≈ 0.86) which we designate the M-state (Figure 2B). The M-state has a higher FRET efficiency than expected from FPS calculations based on the crystal structure. This result is presumably not influence by dye attachment sites, since an inversely labeled construct yields a virtually identical FRET distribution (Supplementary Figure S1).

Upon addition of Gdm^+^ ligand, the equilibrium was shifted to an intermediate FRET efficiency of 0.69 (Figures 2C,D). This FRET value is in excellent agreement with the FPS-modelled distance in the crystal structure of single hairpins in a kissing loop interaction. Therefore, the intermediate FRET conformation is assigned as the K-state. In this experiment the formation of the K-state in the case of 50 mM ligand is seemingly independent of the presence of Mg^2+^. It is possible that these high concentrations of the cationic molecule Gdm^+^ are sufficient to emulate the effects of 5 mM divalent Mg^2+^. However, this might not be the case at higher RNA and/or lower ligand concentrations (Schamber et al., 2021).

To summarize these initial experiments depending on the solution conditions, three distinct FRET states for the guanidine-II riboswitch were identified. For all of these states we verified that the data indeed originated from individual molecules by monitoring single step photobleaching in time resolved experiments (examples shown in Supplementary Figure S2). This strongly suggests that an RNA fold with fluorophore distances as expected for a kissing loop interaction between P1 and P2 is formed *in cis* within a full-length aptamer construct. Further, a previously uncharacterized M-conformation was identified.

### Coarse-Grained Simulations and smFRET Analysis of Mg^2+^-Dependent Folding

**FIGURE 3:**
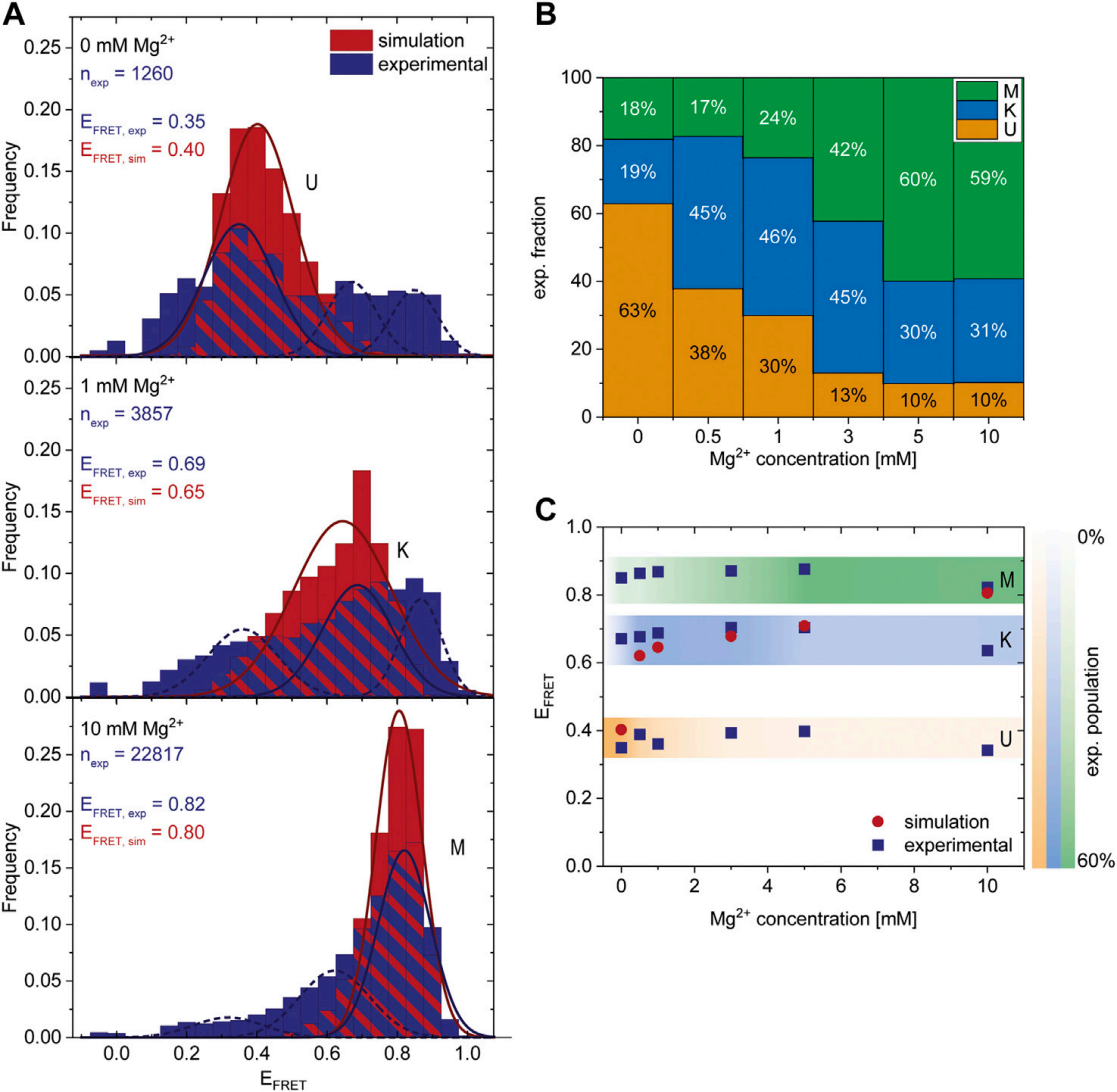
Comparison of experimental smFRET and simulated response of the riboswitch RNA to varying Mg^2+^ concentrations. **(A)** Histograms of the populations obtained in the smFRET experiments (exp) (dark blue) and in the coarse-grained simulations (sim) (red) at different Mg^2+^ concentrations. n_exp_ describes the number of molecules represented in the experimental histograms. The experiments were fitted with three Gaussians corresponding to the three states. The dark blue solid line represents the main state at the conditions, the other states are shown as a dashed line. The simulations were fitted with 1 Gaussian (red line) since at each concentration predominantly one state was populated while the probability of the other states was negligibly small. The maximum of the Gaussian fits E_FRET,exp_ and E_FRET,sim_ corresponds to the FRET efficiency of the most probable structure of each state and is shown as inset. **(B)** Experimental fraction of riboswitches in the three states at various Mg^2+^ concentrations. The fractions were calculated from the area under the fits (shown in **A**). **(C)** Comparison of most probable FRET efficiency E_FRET_ (corresponding to the maxima in the Gaussian fits shown in **A**) from experiments and simulations at various Mg^2+^ concentrations.

After finding a Mg^2+^-dependent state, we investigated the response of the riboswitch to changes in the Mg^2+^ concentration by performing Mg^2+^ titration experiments while monitoring the abundance of each of the three states. The dark blue histograms in Figure 3A show representative Mg^2+^ concentrations with U, K or M being the most dominant peaks, respectively. All other measured FRET histograms can be found in the Supplementary Figure S3. After fitting of these data we calculated the distribution of the three states (Figure 3B) and the mean FRET efficiency of each fit (Figure 3C). We found that high Mg^2+^ concentrations of 5 mM or above lead to stabilization of the M-state in smFRET experiments, while Mg^2+^ concentrations between 0.5 and 3 mM cause a shift from the U- to the K-state (Figure 3B).

**FIGURE 4:**
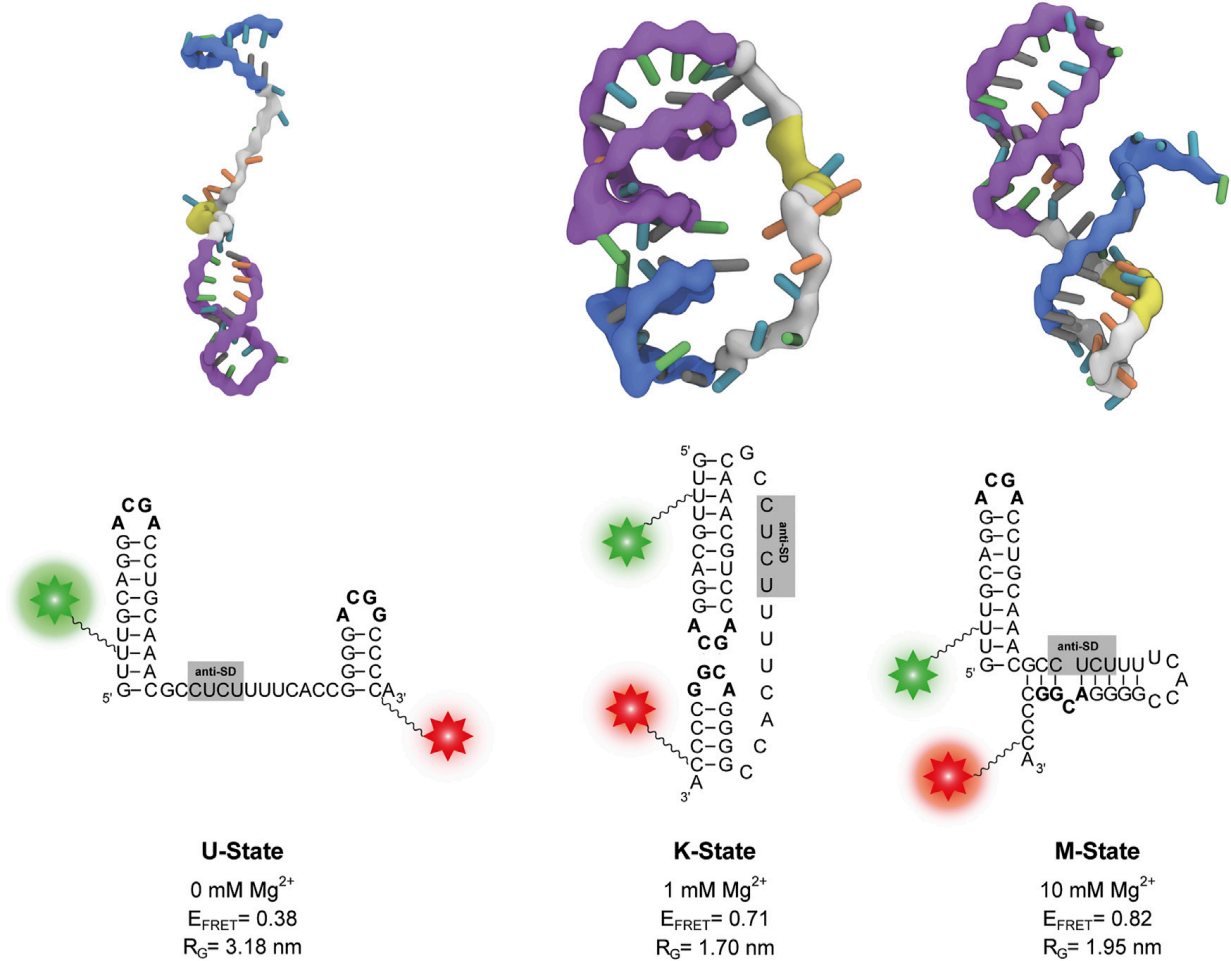
Representative structures of the three states from coarse-grained simulations at different Mg^2+^ concentrations. The P1 and P2 stem-loops are coloured in purple and blue respectively, the anti-SD sequence is coloured in yellow and the rest of the linker is coloured in grey. The corresponding secondary structure is shown below each three-dimensional structure. Positions of Cy3 and Cy5 fluorophores of the smFRET construct are depicted as a green and red star, respectively. The calculated FRET efficiency (E_FRET_) of each structure averaged over different dye orientations is given. R_G_ describes the radius of gyration.

To further characterize these states, we performed coarse-grained simulations at various Mg^2+^ concentrations. In agreement with the experiments, the coarse-grained simulations suggest the existence of three different states dependent on the Mg^2+^. Representative structures of each state were selected from the simulations (Figure 4). The other states are accessible from the K state in these simulations, as evidenced by the time resolved radius of gyration (Supplementary Figure S4).

In absence of Mg^2+^ ions, the linker in the obtained structures is extended. As a result, the P1 and P2 stem-loops are pointing in different directions, and the tetraloop sequences are not in spatial proximity (Figure 4). At 0.5 mM–5 mM Mg^2+^, we find mainly structures with a native-like kissing loop orientation of the P1 and P2 stem-loops (Figure 4). At 10 mM Mg^2+^, more compact structures with a different secondary structure are most abundant. While the P1 hairpin remains folded, the P2 stem unfolds and forms new basepairs with the linker containing the putative anti-SD sequence (Figure 4).

We performed secondary structure predictions with two online tools for an independent assessment. Using the sequence used in this work Mfold v2.3 (Zuker 2003) found a structure with P1 and P2 folded equivalent to the structure at 0 mM Mg^2+^ shown in Figure 4 (Supplementary Figure S5A). When replacing the stabilizing G1 to its native nucleotide additionally to the P1 and P2 conformation another secondary fold is predicted to be more stable by Mfold (Supplementary Figure S5B). This secondary structure is identical to the one obtained from the coarse-grained simulations at 10 mM Mg^2+^ (Figure 4) providing further evidence for the existence of this novel structure. Furthermore, Vienna RNAfold (Lorenz et al., 2011) also predicts this conformation for our guanidine-II riboswitch RNA (Supplementary Figure S5C).

To assess whether the conformations derived from the simulations and the FRET experiments are representatives of the same state, we calculated the theoretical FRET efficiencies and their distributions from the simulations (Figure 3A (red), Supplementary Figures S6, S7). In the coarse-grained simulations, the native K-state is designed to be the minimum energy structure by including the native contacts of the kissing-loop interaction. At the same time, the coarse-grained model includes non-native interactions and therefore allows us to capture the U-state in the absence of Mg^2+^ and the M-state at 10 mM Mg^2+^. Note that in principle different conformations can contribute to the same FRET efficiency and hence to the same state. In particular, in the U-state a variety of different conformations could be distinguished (Supplementary Figure S8), while in the K- and M-state the contributing conformations were similar in structure. Due to the Gō-like nature of the model, the abundance of the conformations in each state does not correspond to an equilibrium distribution. In addition, we find predominantly one conformational state and interconversion between the states within the timescales of our simulation is rare. Therefore, the magnitude of the FRET frequency from simulations and experiments varies as expected (Figure 3A). Moreover, in the coarse-grained simulations a higher Mg^2+^ concentration is required to shift the system since the Gō-like nature of our model stabilizes the K-state.

Still, the FRET efficiencies of the most probable structure in each state from simulations and experiments (E_FRET_) can be compared directly. Figures 3A,C show that E_FRET_ from experiments and simulations is in very good agreement over a range of Mg^2+^ concentrations. This in turn suggests that the structures derived from the coarse-grained simulations and the ones observed in the FRET experiments at different concentrations might be identical, or at least comparable.

These results show that combining coarse-grained simulations and smFRET experiments provides detailed, complementary insights into the response of the riboswitch RNA to different Mg^2+^ concentrations. They also reveal that a coaxial orientation of the hairpins necessary for the kissing loop conformation is accessible in the absence of the ligand. Increasing Mg^2+^ concentrations lead to the folding of the riboswitch into an alternative M-conformation. Here, the coarse-grained simulations allow us to resolve the alternative base pairing pattern and the three-dimensional structure.

### Analysis of Dynamics: Mg^2+^ Titration

**FIGURE 5:**
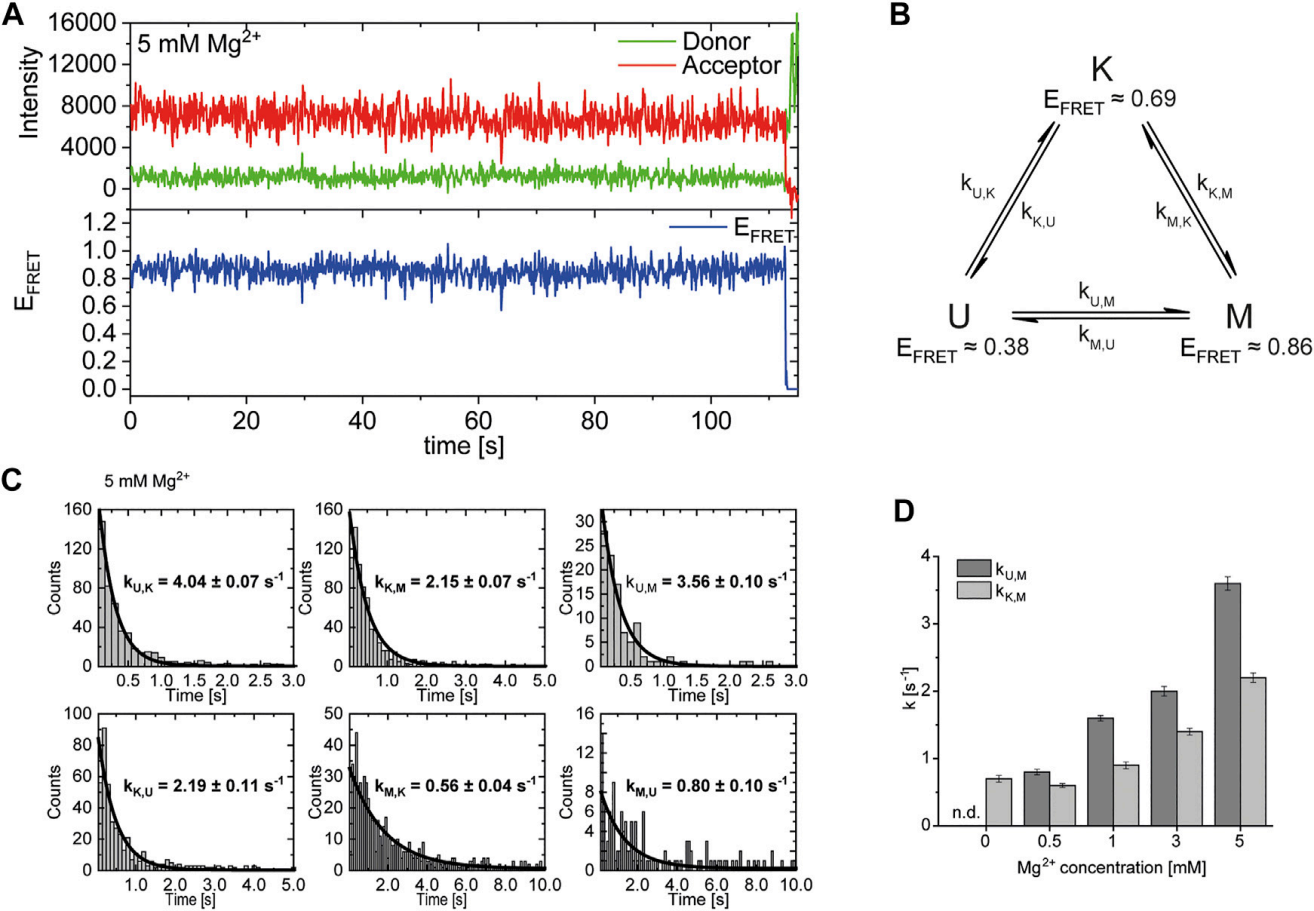
**(A)** Example of a FRET trace of a static molecule in the M-state. **(B)** Three state kinetic model. **(C)** Examples of dwell time plots used to determine transition rates. smFRET trajectories for each condition were fitted for three states with HaMMy and resulting dwell times were fitted with a monoexponential decay. **(D)** Change of transition rates with increasing Mg^2+^ concentration of the transitions of the U to M (dark grey) and K to M (light grey).

After identification and structural description of the three states U, K and M we characterized the transitions between these states. To this end, we performed time resolved smFRET measurements and observed the behavior of the molecules over several minutes at different Mg^2+^ concentrations in the absence of Gdm^+^. While some of the traces remained in one FRET state throughout the measurement (example in Figure 5A), the majority of molecules showed multiple transitions, in which each of the states was accessible directly from every other state. We isolated FRET data prior to photobleaching for each molecule and stitched these traces together to a single FRET trace up to 50,000 datapoints (Supplementary Figure S9). From these stitched traces, we performed hidden Markov Modelling using the HaMMy software (McKinney et al., 2006) with a three state model (Figure 5B). We were able to extract kinetic information (Table1) about the fast-transitioning molecules. For this we plotted the dwell times and fitted a mono-exponential function to derive rate constants (Figure 5C).

**TABLE 1:** Transition rates derived from dwell time fits at various Mg^2+^ concentrations.

Transition	0 mM	0.5 mM	1 mM	3 mM	5 mM
k_U,K_ [s^−1^]	5.6 ± 0.14 (n = 313)	5.8 ± 0.16 (n = 626)	3.2 ± 0.04 (n = 637)	4.6 ± 0.08 (n = 541)	4.0 ± 0.07 (n = 366)
k_K,U_ [s^−1^]	1.1 ± 0.03 (n = 291)	1.0 ± 0.03 (n = 575)	1.6 ± 0.03 (n = 511)	1.5 ± 0.03 (n = 439)	2.2 ± 0.11 (n = 336)
k_U,M_ [s^−1^]	n.d. (n = 21)	0.8 ± 0.04 (n = 423)	1.6 ± 0.04 (n = 339)	2.0 ± 0.07 (n = 221)	3.6 ± 0.10 (n = 110)
k_M,U_ [s^−1^]	n.d. (n = 43)	0.8 ± 0.04 (n = 476)	0.6 ± 0.03 (n = 466)	0.8 ± 0.05 (n = 324)	0.8 ± 0.10 (n = 140)
k_K,M_ [s^−1^]	0.7 ± 0.05 (n = 73)	0.6 ± 0.03 (n = 168)	0.9 ± 0.05 (n = 367)	1.4 ± 0.05 (n = 315)	2.2 ± 0.07 (n = 272)
k_M,K_ [s^−1^]	n.d. (n = 51)	1.2 ± 0.03 (n = 115)	0.7 ± 0.04 (n = 240)	0.4 ± 0.03 (n = 212)	0.6 ± 0.04 (n = 243)

In cases where a reliable fit could not be obtained, i.e., due to an insufficient number of transitions (n), the rates were not determined (n.d.).

We found that the transition from the U-state to the K-state was fast, with a rate constant of approximately k_U,K_ = 4.6 s^−1^ (average over all Mg^2+^ concentrations). We note that this transition is a reorientation of the two hairpins P1 and P2 rather than opening of any helix, as suggested by the structures from the coarse-grained simulations. For opening the kissing loop, we found slightly slower rates of k_K,U_ between 1.0 and 2.2 s^−1^. For the formation of the M-conformation a likely Mg^2+^-dependent behavior from both the U- and the K-state was observed, in which rates increased slightly with Mg^2+^ concentration (Figure 5 and Table 1). This increase ranged from 0.8 to 3.6 s^−1^ for k_U,M_ and from 0.6 to 2.2s^−1^ for k_K,M_. Refolding into both the U- and K-state starting from the M-state occurred slower in comparison, with no obvious dependence on Mg^2+^ concentrations for K and U (around k_M,U_ = 0.8 s^−1^ and k_M,K_ = 0.7 s^−1^). This slow refolding was also observable in the small number of molecules in the timescale of this analysis especially for the M-state to K-state transition which resulted in limited resolution of the corresponding fits. It is worth noting that transitions from the unfolded conformation into the kissing loop conformation occur on a timescale similar to unfolding of the Mg^2+^-dependent conformation, despite the latter requiring release of several basepairs within a helix. One possible explanation is that folding into the K-state requires a significant entropic contribution associated with restricting conformational degrees of freedom of the U-state.

### Effect of Gdm^+^ Ligand on Structural Dynamics

**FIGURE 6:**
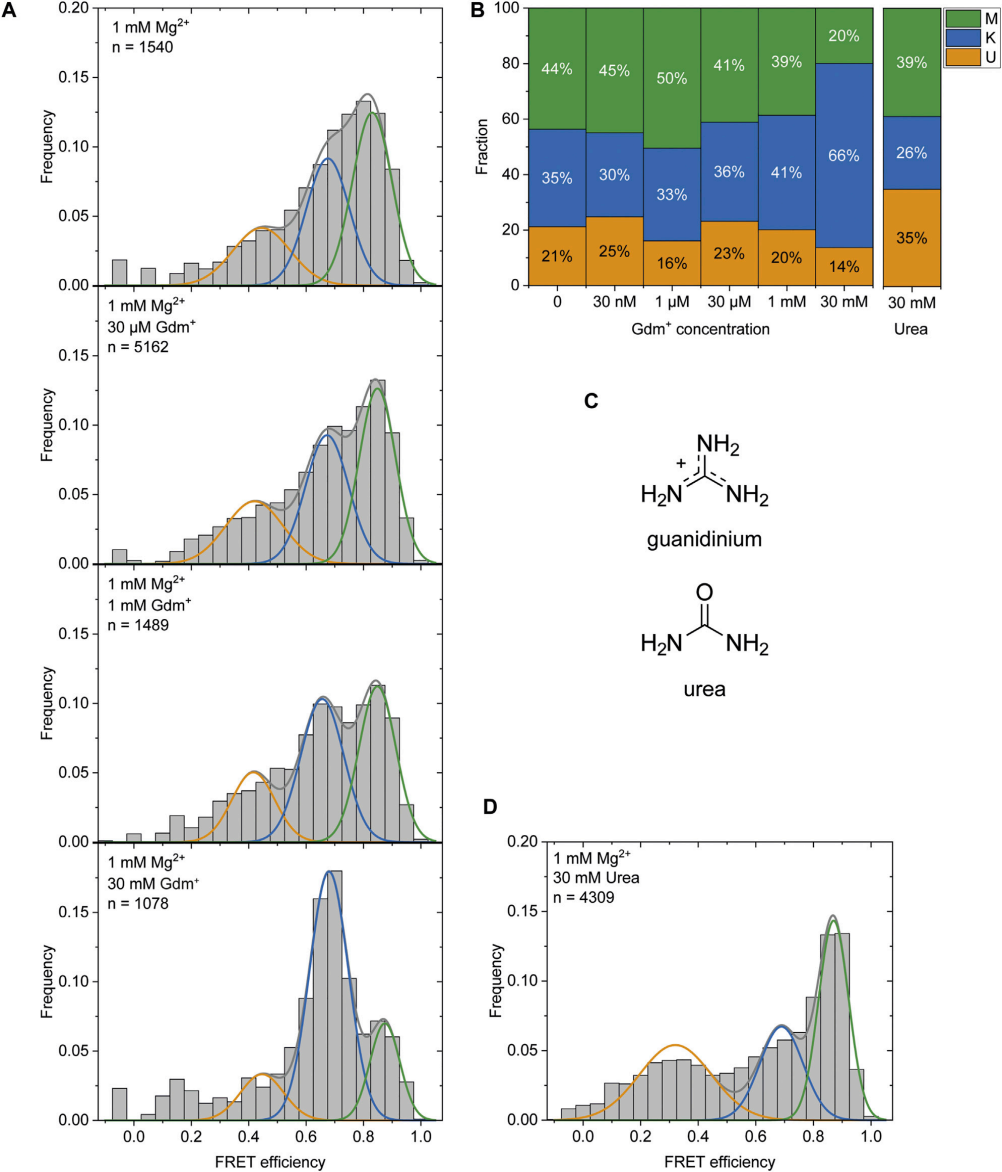
**(A)** Histogram analysis of the Gdm^+^ titration at constant 1 mM Mg^2+^. The data were fitted with three Gaussian fits showing the change between the K-state (blue) and the M-state (green) with increasing Gdm^+^ concentration. **(B)** Fractions of molecules adopting the individual states based on the area under the fitted curves. **(C)** Structures of Gdm^+^ and urea. **(D)** Histogram analysis at 1 mM Mg^2+^ with 30 mM urea.

It was previously reported that Gdm^+^ acts as the ligand for this riboswitch class and leads to a kissing loop formation (K-conformation) upon binding to the loops of the P1 and P2 hairpins (Sherlock et al., 2017). After observing the K-state to a certain amount (46% at 1 mM Mg^2+^) in the absence of the ligand and a stabilization of this conformation (to 66%) at high Gdm^+^ concentrations of 50 mM (Figure 2), we characterized the ability of P1 and P2 to form a kissing loop orientation in a ligand dependent manner. We chose the intermediate (and likely physiological) Mg^2+^ concentration of 1 mM (Tyrrell et al., 2013) to perform ligand titrations. Increasing Gdm^+^ concentrations in the sub-millimolar range did not result in significant shifts between the populations, with 30–41% of the molecules adopting the K-state. Addition of 30 mM Gdm^+^ however shifted the populations into the K- state (66%) on the expense of both U and M states, confirming a distinct ligand dependence of this conformation (Figures 6A,B and Supplementary Figure S10).

The high concentrations of Gdm^+^ used in this experiment could however also have an unspecific chaotropic effect. To exclude this possibility we therefore repeated this measurement in the presence of 30 mM urea (Figure 6C) instead of Gdm^+^ to further assess the specificity of ligand binding. In contrast to Gdm^+^, the same concentrations of urea did not shift the equilibrium towards the K-state, but rather resulted in a destabilization of the RNA as evidenced by an increased and likely broadened U-state population (35% of molecules) (Figure 6D).

**FIGURE 7:**
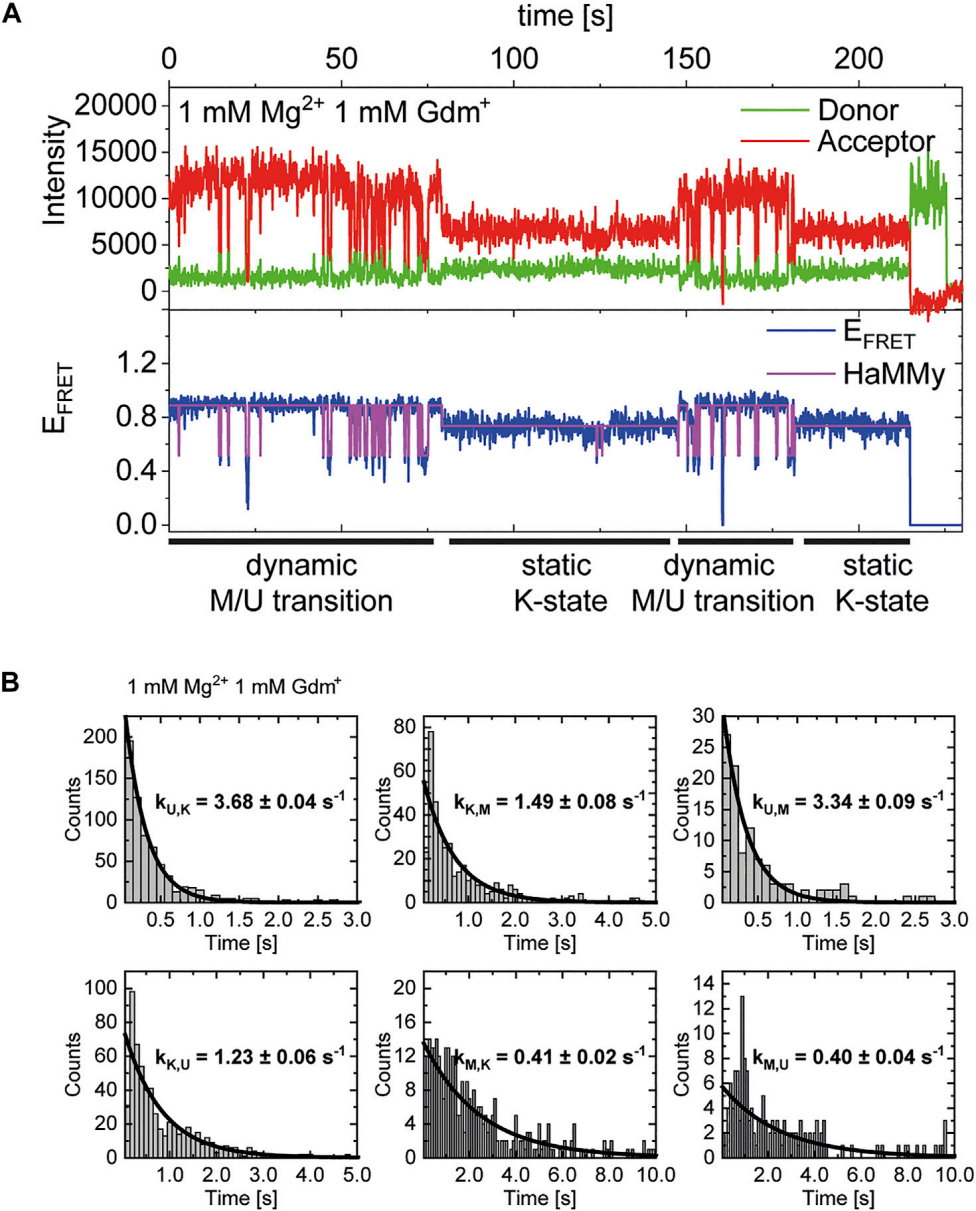
**(A)** Example of time-resolved FRET behavior of an interconverting molecule switching between a dynamic M-state and a static K-state at constant 1 mM Mg^2+^ and 1 mM Gdm^+^. **(B)** Examples of dwell time plots of 1 mM Mg^2+^ and ligand each, used for transition rate calculations.

In addition to our histogram analysis, we performed experiments to assess the time-resolved FRET behavior of the riboswitch RNA at 1 mM Mg^2+^ at different ligand concentrations. Again, we found molecules that are interconverting between the three FRET states as shown for one representative example molecule in Figure 7A, which switches between a dynamic M-state and a static K-state. As for the Mg^2+^ dependence we also calculated the rate constants for all observable transitions in our Gdm^+^ titration (Figure 7B and Table 2) from the dwell times derived from stitched traces (Supplementary Figure S11). Here, transitions between the U- and K-states are comparable with the data obtained from Mg^2+^ titration experiments, i.e. with k_U,K_ = 3.9 s^−1^ and k_K,U_ = 1.3 s^−1^, respectively. In general, all other observed transition rates were also comparable with the data of the Mg^2+^ titration in absence of ligand. Within error, we were not able to identify a faithful ligand dependence of any of the fast transitions, with the exception of k_U,K_, which upon increase of the ligand concentration rose from ≤3.7 s^−1^ up to 1 mM Gdm^+^ to 6.4 s^−1^ at 30 mM Gdm^+^.

**TABLE 2:** Transition rates derived from dwell time fits at various Gdm^+^ concentrations at 1 mM Mg^2+^.

Transition	0	30 nM	1 µM	30 µM	1 mM	30 mM
k_U,K_ [s^−1^]	3.2 ± 0.04 (n = 737)	3.5 ± 0.04 (n = 730)	2.9 ± 0.04 (n = 929)	3.7 ± 0.07 (n = 934)	3.7 ± 0.04 (n = 682)	6.4 ± 0.09 (n = 933)
k_K,U_ [s^−1^]	1.6 ± 0.03 (n = 729)	1.0 ± 0.05 (n = 696)	0.8 ± 0.05 (n = 903)	1.9 ± 0.03 (n = 846)	1.2 ± 0.06 (n = 632)	1.4 ± 0.03 (n = 930)
k_U,M_ [s^−1^]	1.6 ± 0.04 (n = 79)	2.4 ± 0.11 (n = 95)	1.5 ± 0.05 (n = 917)	1.4 ± 0.05 (n = 215)	3.3 ± 0.09 (n = 107)	5.0 ± 0.19 (n = 49)
k_M,U_ [s^−1^]	0.6 ± 0.03 (n = 86)	0.3 ± 0.04 (n = 129)	0.3 ± 0.03 (n = 223)	0.4 ± 0.03 (n = 303)	0.4 ± 0.04 (n = 157)	0.6 ± 0.11 (n = 51)
k_K,M_ [s^−1^]	0.9 ± 0.05 (n = 71)	0.8 ± 0.05 (n = 143)	0.7 ± 0.06 (n = 162)	1.0 ± 0.07 (n = 428)	1.5 ± 0.08 (n = 402)	n.d. (n = 26)
k_M,K_ [s^−1^]	0.7 ± 0.04 (n = 64)	0.2 ± 0.03 (n = 109)	0.2 ± 0.02 (n = 135)	0.3 ± 0.02 (n = 340)	0.4 ± 0.02 (n = 353)	n.d. (n = 23)

In cases where a reliable fit could not be obtained, i.e. due to a low number of transitions (n), the rates were not determined (n.d.).

This analysis can solely characterize fast dynamic molecules and does not include slow refolding or molecules with a stable conformation (e. g., ligand stabilization) due to the timescales accessible in our experiments. As can be seen in Figure 7A, we do however observe some molecules that transiently adopt the K-state for an extended period of time (>∼20 s). In addition to that, all of the data sets contained a limited number of molecules that adopted a particular state (U, K, or M) and showed no transitions between states for the duration of the observation (termed “static molecules”). Therefore, we cannot definitively state whether the molecules used for the fast dynamics in Table 2 necessarily have a Gdm^+^ molecule bound.

## Discussion

Using retrosynthetic splitting, we successfully designed and synthesized a smFRET construct for analysis of individual guanidine-II riboswitch RNA aptamer domain molecules. This RNA showed a distinct and reversible response to binding of both Mg^2+^ and Gdm^+^ ions and folding into three discernible states (Unfolded, Kissing loop orientation, and Mg^2+^ dependent) in both smFRET experiments and coarse-grained simulations.

In our smFRET experiments we find an intermediate FRET efficiency state (K-state). This FRET state is accessible without ligand but is strongly stabilized with increasing Gdm^+^ as would be expected for the kissing interaction forming RNA fold according to literature (Huang et al., 2017; Reiss and Strobel 2017; Sherlock et al., 2017). FRET value prediction derived from FPS-based modelling of the crystal structure (Huang et al., 2017) closely matches our experimental data. This strongly supports that we observe a kissing loop conformation in form of the K-state. Since our methodological approach can robustly identify signals from individual molecules via single molecule photobleaching, this is a very strong indication that the interaction between P1 and P2 helices can indeed occur *in cis*. For further support we conducted coarse-grained simulations in absence of the Gdm^+^ ligand. At 0.5 mM–3 mM Mg^2+^, where the K-state is the most populated in smFRET measurements, the simulated molecules also fold into the kissing loop orientation. In addition to the structural description, experimental FRET values of the K-state and those calculated from the simulated structures are in good agreement, even though several factors affect accuracy of these smFRET-derived distances (Lerner et al., 2021). In remarkable consistence within the Mg^2+^ titration it showcases how a combined approach of smFRET and coarse-grained simulations can link FRET distributions to structural information.

In the model of guanidine-II riboswitch regulation with an opening and closing of the kissing loop (Figure 1A) a larger distance of the hairpins is expected in an open conformation in the absence of ligand. smFRET data indeed suggest an RNA fold with a larger distance than the K-state, as evidenced by a low FRET efficiency in an environment absent of both Mg^2+^ and Gdm^+^ (U-state). Coarse-grained simulations confirm this FRET state to be the open conformation with consistency of theoretical to experimental FRET efficiencies under the same conditions. It furthermore structurally characterizes the open U-RNA conformation with the formation of both hairpins P1 and P2, but no interaction between them, but rather separated by an extended linker.

Using smFRET, we also experimentally describe the dynamic behaviour of the guanidine-II riboswitch. Fast transitions between the FRET states that were identified as the kissing interaction (K-state) and the open conformation (U-state) are found. In these, rates from the K- to the U-state are slightly slower than from U- to K-state. This is in agreement with possible hydrogen bonds between the loop CGs of both hairpins being formed even in transient conformations. This leads to the conclusion that we are able to observe the reorientation of the pre-formed hairpins toggling around the flexible linker. No unambiguous Gdm^+^ dependence was detected for the transition kinetics of this freely moving RNAs. Even though smFRET is not able to distinguish whether the molecules accessible for dynamics analysis have ligand bound, titration of Gdm^+^ results in a stepwise ligand-dependent stabilization of the kissing state in FRET histograms. In addition to dynamically reorienting molecules some other molecules adopt a stable FRET state for an extended period of time. The change in FRET histograms with increasing Gdm^+^ concentrations might be explained with a higher fraction of molecules stabilized in a static K-state temporarily losing the ability of reorientation. For ligand binding, an increase of folding from the U into the K state requires very high (30 mM) concentration of ligand. While the K_d_ for this RNA is generally high [300 µM (Sherlock et al., 2017)], this points to an even higher concentration that is required for full stability of the kissing loop interaction. Since we also observe the kissing orientation in absence of the native ligand Gdm^+^, we cannot definitively identify whether Gdm^+^ ligand binding induces the kissing loop interaction (“induced fit”), or whether binding of the ligand merely stabilizes molecules that have already adopted a kissing loop orientation (“conformational selection”).

Unexpected from any published model of the guanidine-II class riboswitch, further increasing the Mg^2+^ concentration in our smFRET Mg^2+^ titration in absence of ligand leads to an even higher FRET efficiency than the K-state. This state is kinetically favored by Mg^2+^ with increasing rate constants of M-state formation with increasing Mg^2+^. Interestingly, the structure described by the simulations at 10 mM Mg^2+^ concentrations is fully consistent with the measured FRET values even in absence of any other, orthogonal *a priori* structural knowledge furthermore underlining the importance of a combined methodological approach. Nevertheless, this conformation is supported by independent secondary structure prediction. The M-conformation exhibits a distinctly different secondary structure and base pairing pattern for the shorter P2 hairpin, binding back to the linker with the P1 formed natively. The slow rate constants exiting this state could in principle be rationalized by these structural information as it presumably requires breaking eight base pairs. Taken together we characterize a novel Mg^2+^-dependent RNA fold that is in equilibrium with the U- and K-conformation. These results suggest that the RNA requires a certain window of Mg^2+^ concentrations that facilitate folding into the kissing loop structure in order to be able to respond to environmental influences.

The M-structure is also in agreement with other data from in-line probing experiments (Sherlock et al., 2017), which at high (20 mM) Mg^2+^ concentrations show a rather low cleavage intensity of the P2 loop for the *E. coli* specific RNA even in absence of ligand compared to cleavage of its P1 loop or P2s of other representatives of the guanidine-II riboswitch class. In this study, the authors found increased cleavage of the linker after kissing loop formation. The in-line probing in absence of Gdm^+^ likely reports an equilibrium between U- and M-conformation rather than only the M-structure, which would be consistent with the equilibrium in the smFRET experiments reported here. Differences in cleavage intensities, i.e. for the P1 and P2 stems, in in-line probing data for the *E. coli* riboswitch could therefore also be explained with the structural information from our simulations. The Mg^2+^-dependent RNA structure may be a feature specific to the sequence of this riboswitch aptamer in *E. coli,* and therefore emphasizes the importance of a comparison of data points obtained at different Mg^2+^ concentrations. Since the state occurs at elevated Mg^2+^ concentrations and to a smaller extent also at near-physiological Mg^2+^ concentrations, an unambiguous judgment on a possible regulatory relevance *in vivo* of this structure is not possible. As can be seen in Figure 4, the anti-SD-sequence would be inaccessible in this conformation, since it is interacting with a part of the sequence that is otherwise involved in formation of the P2 hairpin.

With regard to the Mg^2+^ titration, a consistent picture emerges from our combined approach of smFRET experiments and coarse-grained simulations. While smFRET experiments are routinely employed to characterize the Mg^2+^-dependent folding of RNAs (Lambert et al., 2005; Steiner et al., 2008; Kobitski et al., 2011; Hengesbach et al., 2012; Bisaria et al., 2016) and in particular riboswitches (Manz et al., 2017; Warhaut et al., 2017; McCluskey et al., 2019; Warnasooriya et al., 2019), the combined approach used here allows us to provide a more comprehensive view. The quantitative agreement between the maximum efficiencies of each state from simulations and experiments highlights the compatibility of both approaches despite possible limitations in the time scale accessible to the simulations. In particular, the simulations complement the experiments by providing molecular insight into the base pairing pattern and the three-dimensional structures at different Mg^2+^ concentrations.

The comparison of simulations and smFRET data at different Mg^2+^ concentrations and the presence of the M-state also show that a near-physiological range of Mg^2+^ ion concentrations (Tyrrell et al., 2013) is required for the capacity for efficient folding into the suggested functional K state. The K state in turn is significantly stabilized by ligand binding, confirming the functional relevance of our analysis. This also for the first time experimentally directly demonstrates that the functionally relevant interaction between the two hairpins P1 and P2 occurs *in cis* in a ligand-dependent manner.

The integration of single-molecule and simulation data presented in this study has several limitations. Most prominent, as noted above, are the different timescales accessible to the two methods. While FRET experiments are possible in the (sub-)microsecond range (Schuler and Hofmann 2013; Gansen et al., 2018), simulation time is limited by computing time. This is remedied by using models with lower resolution and coarse-grained models are particularly suited to investigate large conformational changes that are out of reach for atomistic simulations. Still, the accuracy of the predictions can be limited by shortcomings of the empiric interaction potential of the model. A close comparison to experimental data, as presented here, is therefore particularly useful to validate coarse-grained models and to assess possible limitations. At the same time, the slow (second timescale) conformational changes observed by smFRET are certainly inaccessible to straight forward simulations techniques. Conversely very fast dynamics can be closely followed using simulations while they would inevitably be averaged out during standard smFRET measurements. Using restraints from only one FRET pair (i.e., using only one distance vector) limits the information available for structural analysis of any given molecule. This is hampered even further by several assumptions dealing with uncertainties in calculation of FRET efficiencies (Lerner et al., 2021). For this very reason, it is impossible to decipher any structure solely from FRET restraints. In several exemplary studies, this is usually mitigated by either additional labeling sites (Stone et al., 2007; Hengesbach et al., 2012; Warhaut et al., 2017; St-Pierre et al., 2021), the use of ligand-specific conformational changes, and construct size. On the spectroscopic side, FRET is inherently limited by several experimental uncertainties, including dye mobility (Iqbal et al., 2008; Schmidt et al., 2018). This also translates into assumptions that go into the treatment of dye linkers for FPS (Kalinin et al., 2012; Dimura et al., 2016) or coarse-grained simulations. Finally, the concentrations used in single-molecule experiments may not be compatible with structural biology approaches or simulations (Schamber et al., 2021), as can be seen in the experiments described above.

In summary, combining coarse-grained simulations and experiments has proven particularly useful to investigate conformational changes in biomolecules (Koculi et al., 2007). The coarse-grained model by Hyeon and Thirumlai (Hyeon and Thirumalai 2005) is particularly suited to investigate large conformational changes of the guanidine riboswitch in response to changes in the Mg^2+^ concentration. On the other hand, smFRET experiments give complementary information and are well established to investigate conformational changes in response to ligand binding and buffer conditions (Holmstrom et al., 2014; Vieweger et al., 2015; Yadav et al., 2022). The combined approach presented here allowed us to validate the coarse-grained model further and to complement the FRET experiments by structural insights into the conformational changes.

## Conclusion

In summary, smFRET analysis and simulations of the full-length guanidine-II riboswitch aptamer domain from *E. coli* shows that the RNA can adopt three distinct states which are responsive to ligand as well as to Mg^2+^ concentration. In close agreement between single molecule experiments and coarse-grained simulations, we find three interconvertible states: An unfolded, open state, a novel Mg^2+^-dependent state, and the presumably functional kissing loop interaction state.

Our results show that combining coarse-grained simulations and single-molecule FRET experiments provides complementary and detailed insights into the conformational changes induced by environmental conditions.

In light of the proposed translation regulation properties of this riboswitch, our findings are also in excellent agreement with existing data from other methodological approaches (Huang et al., 2017; Reiss and Strobel 2017; Sherlock et al., 2017; Wuebben et al., 2020). Our results further provide the first direct evidence of the ligand-dependent kissing loop orientation *in cis* for the guanidine-II riboswitch.

## Data Availability

The raw data supporting the conclusions of this article will be made available by the authors, without undue reservation.
