# Supplementary material for: Combining Coarse-Grained Simulations and Single Molecule Analysis Reveals a Three-State Folding Model of the Guanidine-II Riboswitch
Source: Front Mol Biosci. 2022 Apr 19;9:826505. doi: 10.3389/fmolb.2022.826505 (PMC9094411; doi:10.3389/fmolb.2022.826505)
Supplement: Supplementary file 1 [file DataSheet1.PDF]

Supplementary Material:

Combining coarse-grained simulation and single molecule analysis reveals a three-state folding model of the Guanidine-II riboswitch

Christin Fuks, Sebastian Falkner, Nadine Schwierz, Martin Hengesbach

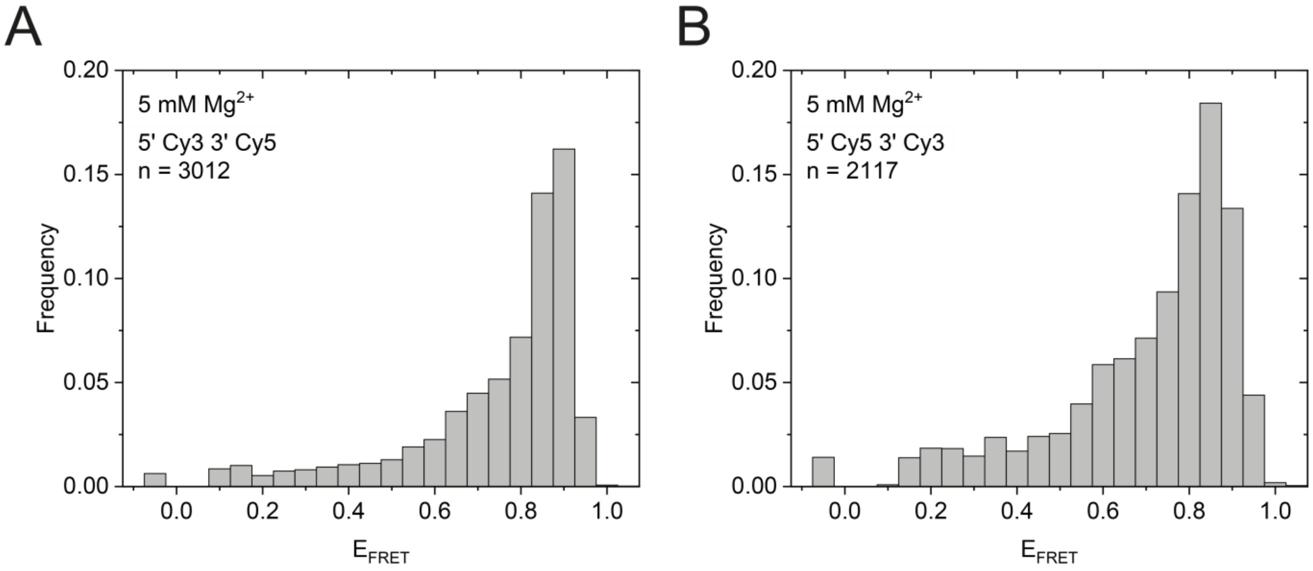

Supplementary Figure S1. FRET histogram at 5 mM Mg^{2+} comparing A) the construct used in this study with a Cy3 label at position U3 and a Cy5 label at the 3' phosphate (5' Cy3 3' Cy5) with B) an inversely labeled construct (5' Cy5 3' Cy3).

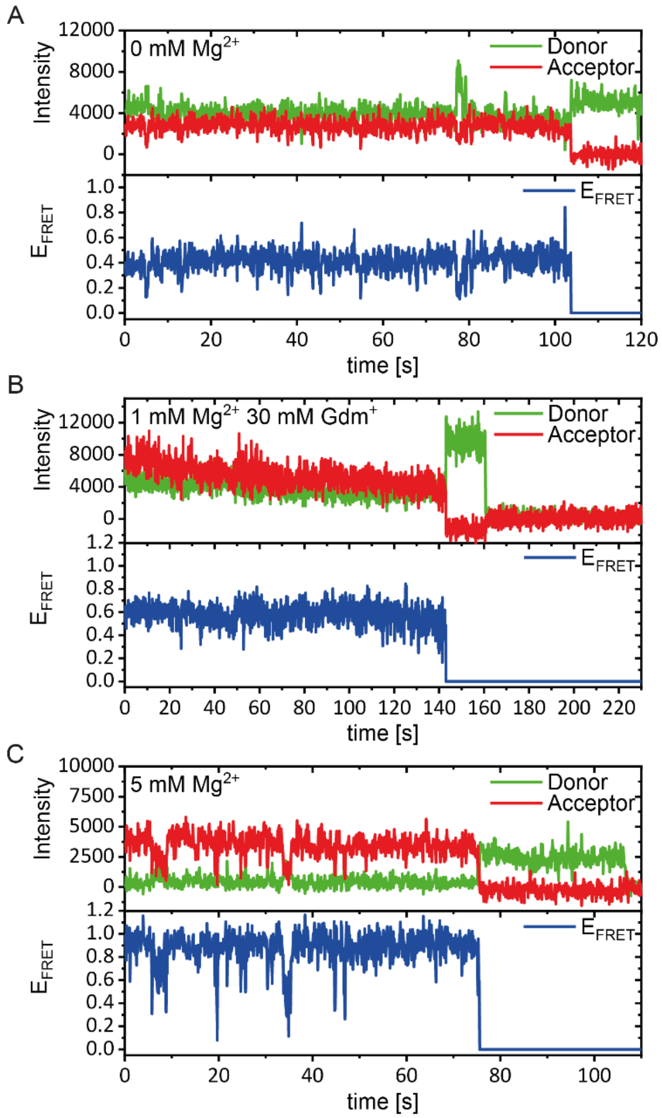

Supplementary Figure S2. Examples for time-resolved smFRET traces showing single step photobleaching of the acceptor for (A) the U-state, (B) the K-state and (C) the M-state.

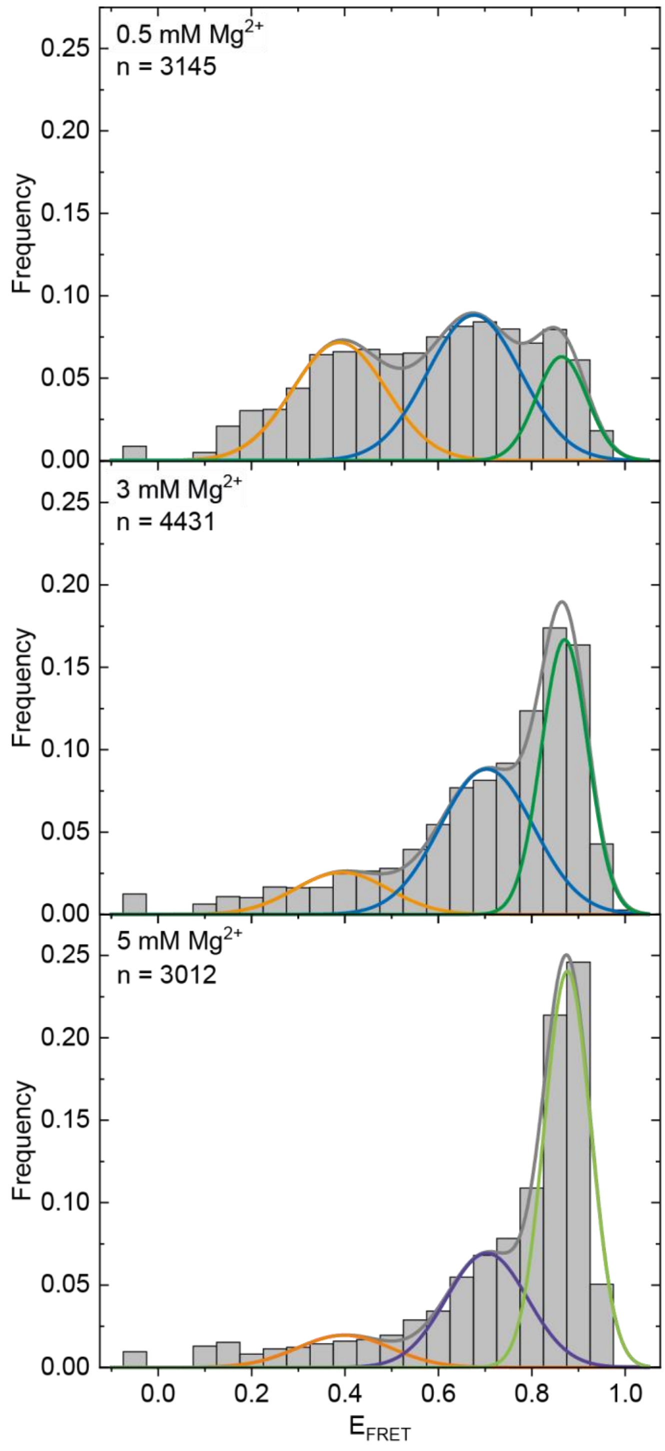

Supplementary Figure S3. Additional experimental smFRET data from the Mg^{2+} titration in absence of ligand fitted with 3 Gaussian fits.

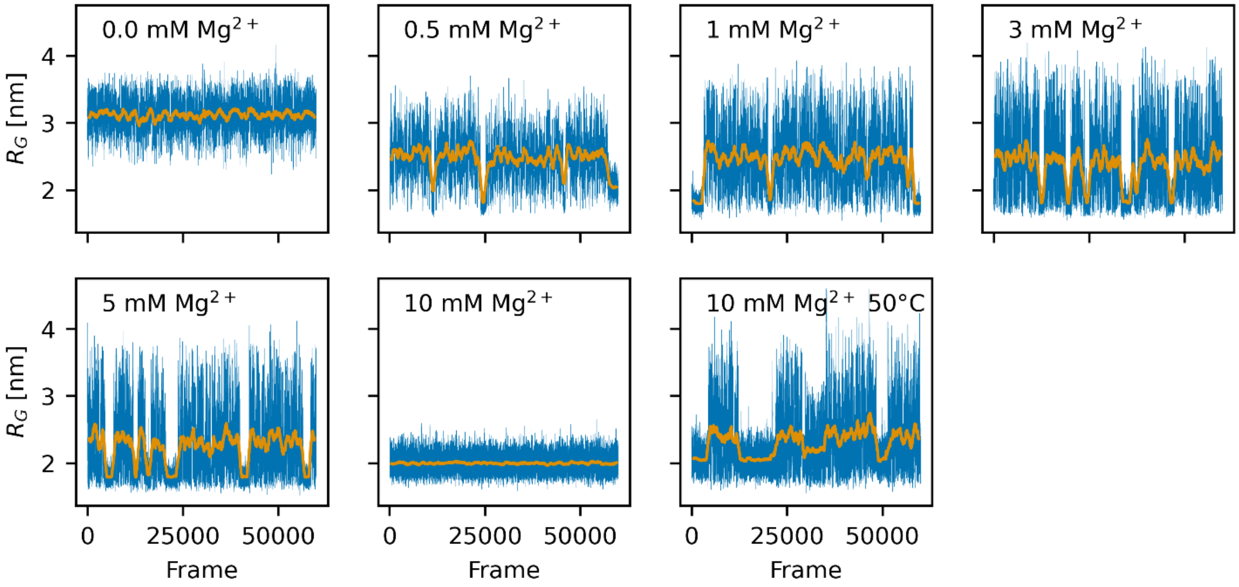

Supplementary Figure S4: Time-resolved radius of gyration at different Mg^{2+} concentrations from coarse-grained simulations.

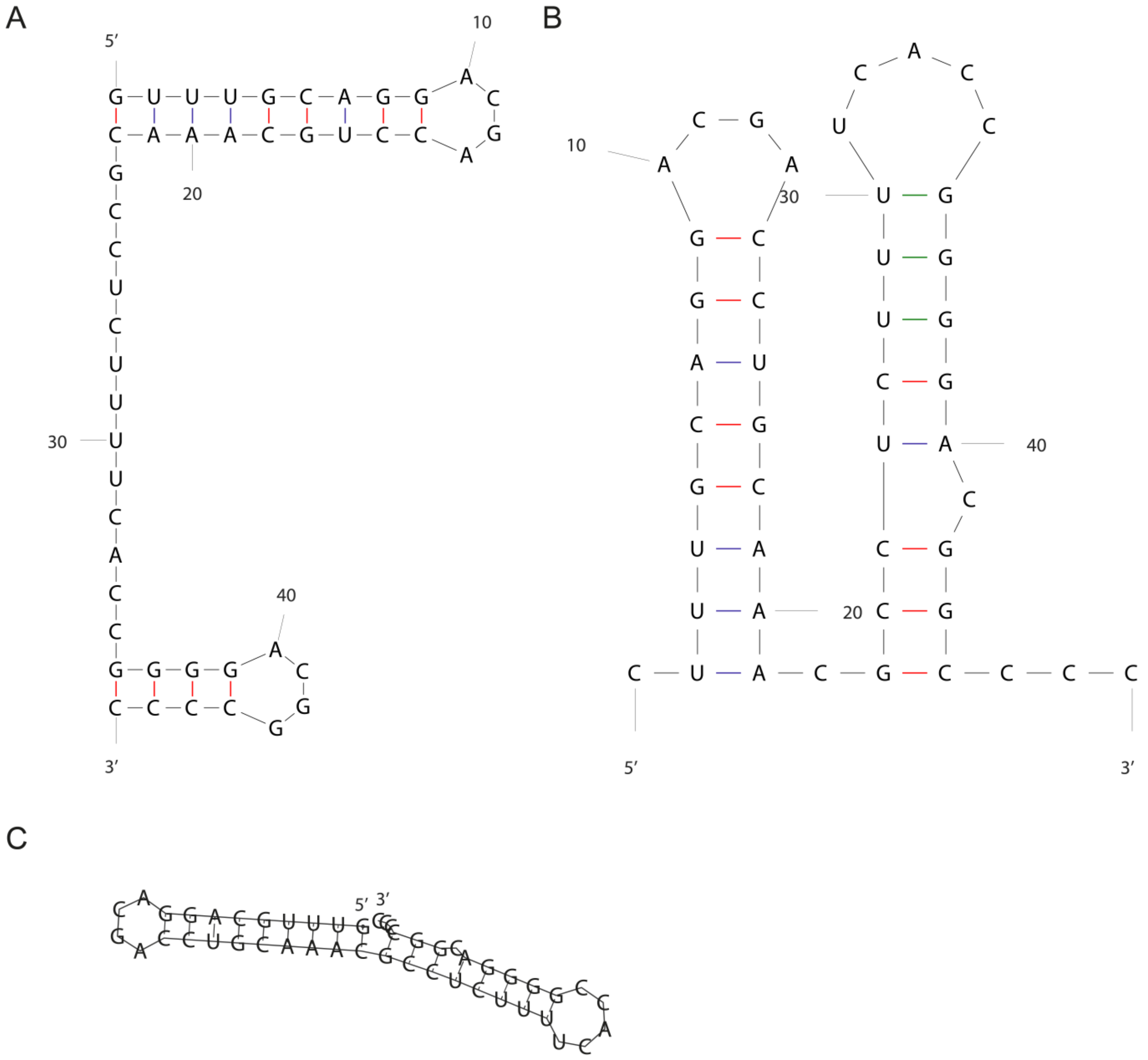

Supplementary Figure S5. Secondary structure prediction of the guanidine-II riboswitch aptamer. (A) Mfold RNA folding Form Version 2.3 predicts the U-conformation with native P1 and P2 as only structure for the 47mer RNA sequence used in this study. (B) Replacing the G1 of the sequence which was introduced for stability with its native nucleotide C Mfold predicts the M-conformation as most stable RNA fold additionally to the U-conformation. (C) Vienna RNAfold predicts the aptamer to fold into the M-conformation.

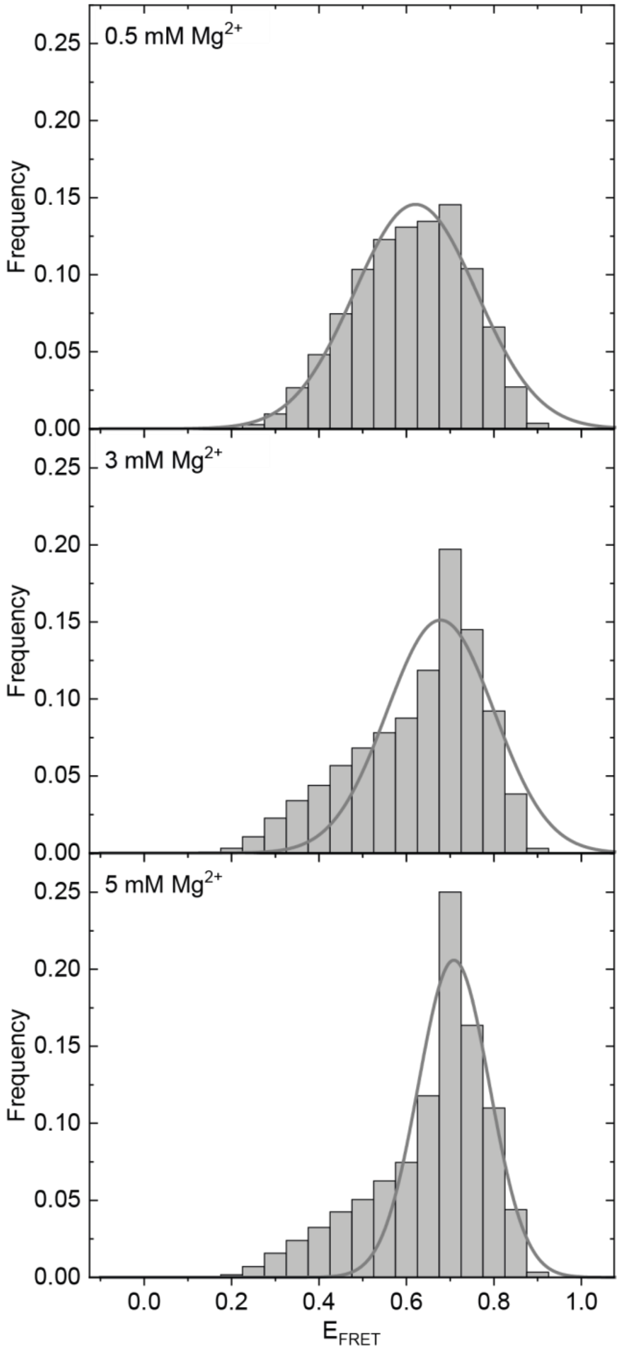

Supplementary Figure S6. Additional histograms derived from coarse-grained simulations from the Mg^{2+} titration fitted with 1 Gaussian fit.

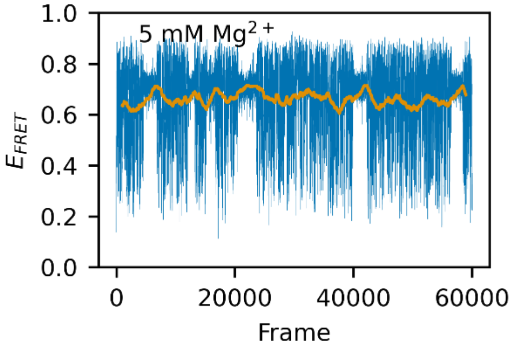

Supplementary Figure S7. Time-resolved FRET efficiency derived from coarse-grained simulations at 5 mM Mg^{2+}.

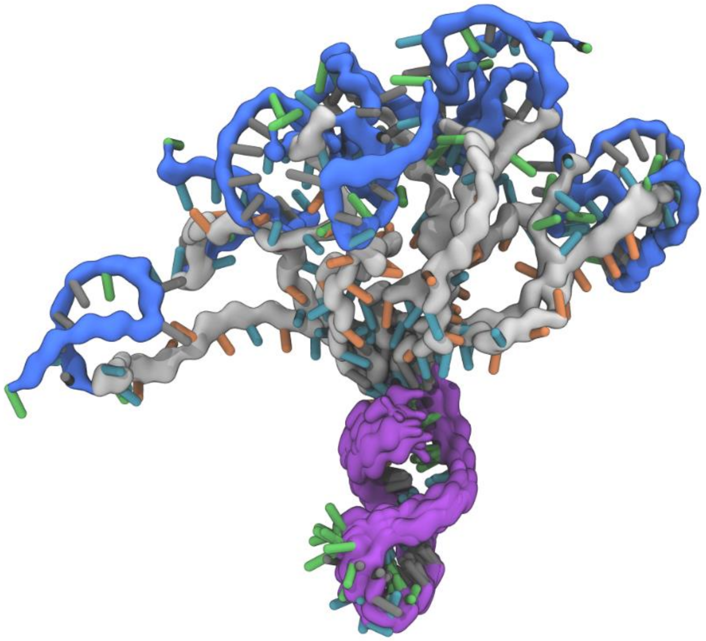

Supplementary Figure S8. Ensemble of different conformations in the U-state from coarse-grained simulations.

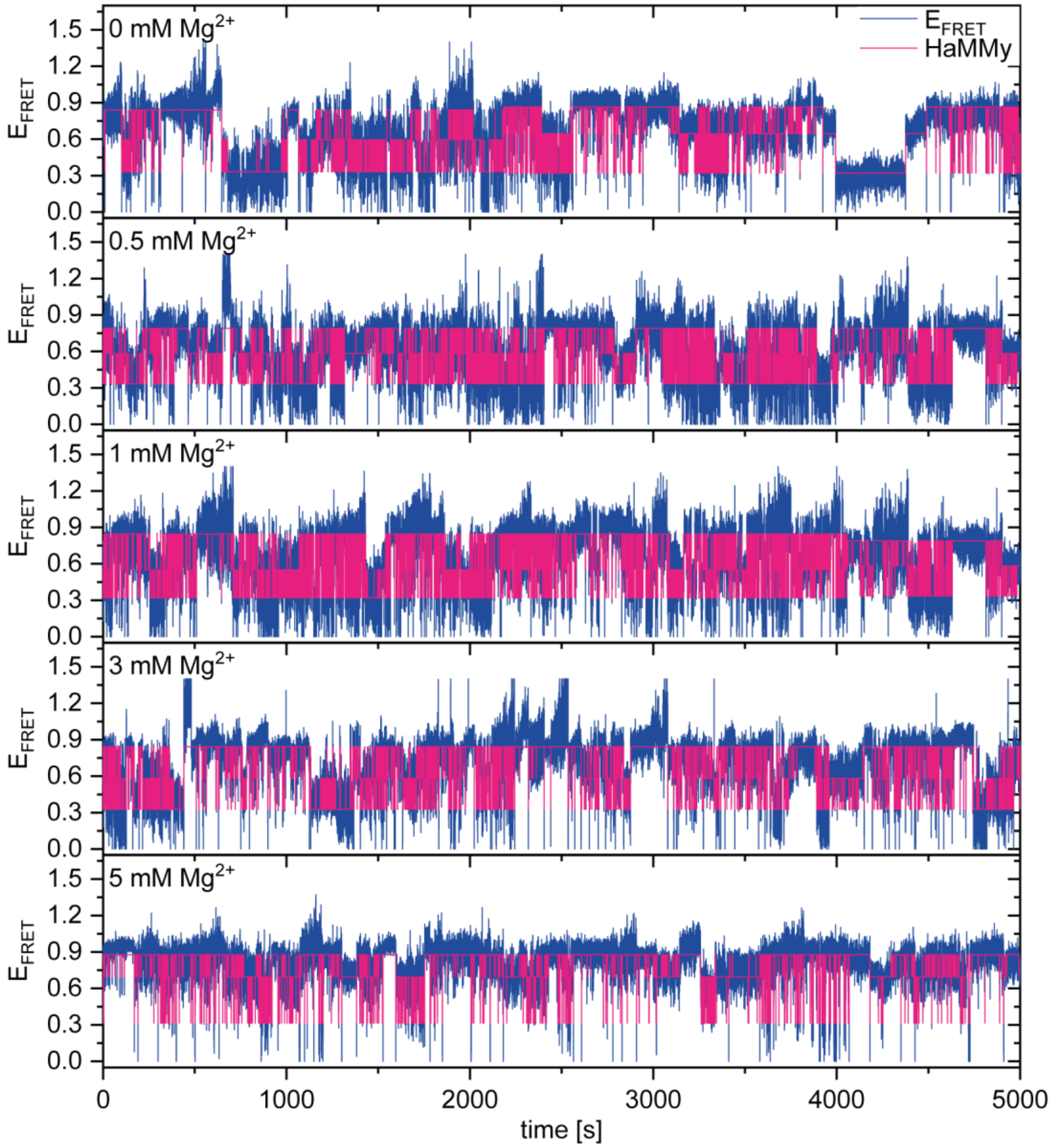

Supplementary Figure S9. Stitched and HaMMy fitted smFRET traces from the Mg^{2+} titration used for kinetic analysis. FRET trajectories were selected manually and stitched to a single trace with 50000 datapoints (blue). Each trace was subjected to a 3 state Hidden Markov modelling using HaMMy (pink).

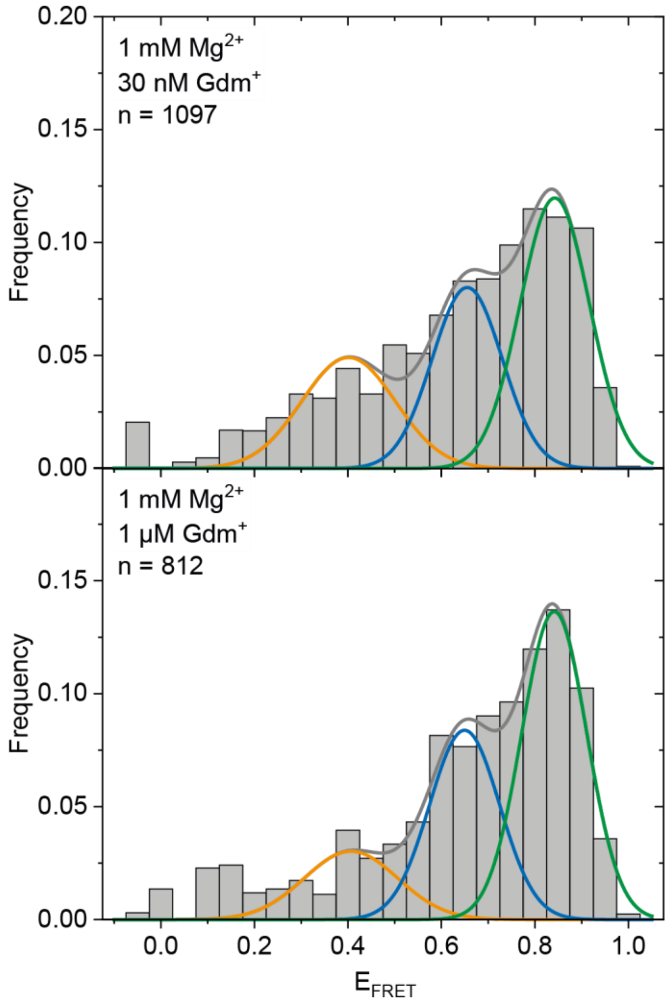

Supplementary Figure S10. Additional experimental smFRET data from the Gdm^{+} titration at constant Mg^{2+} concentration of 1 mM fitted with 3 Gaussian fits.

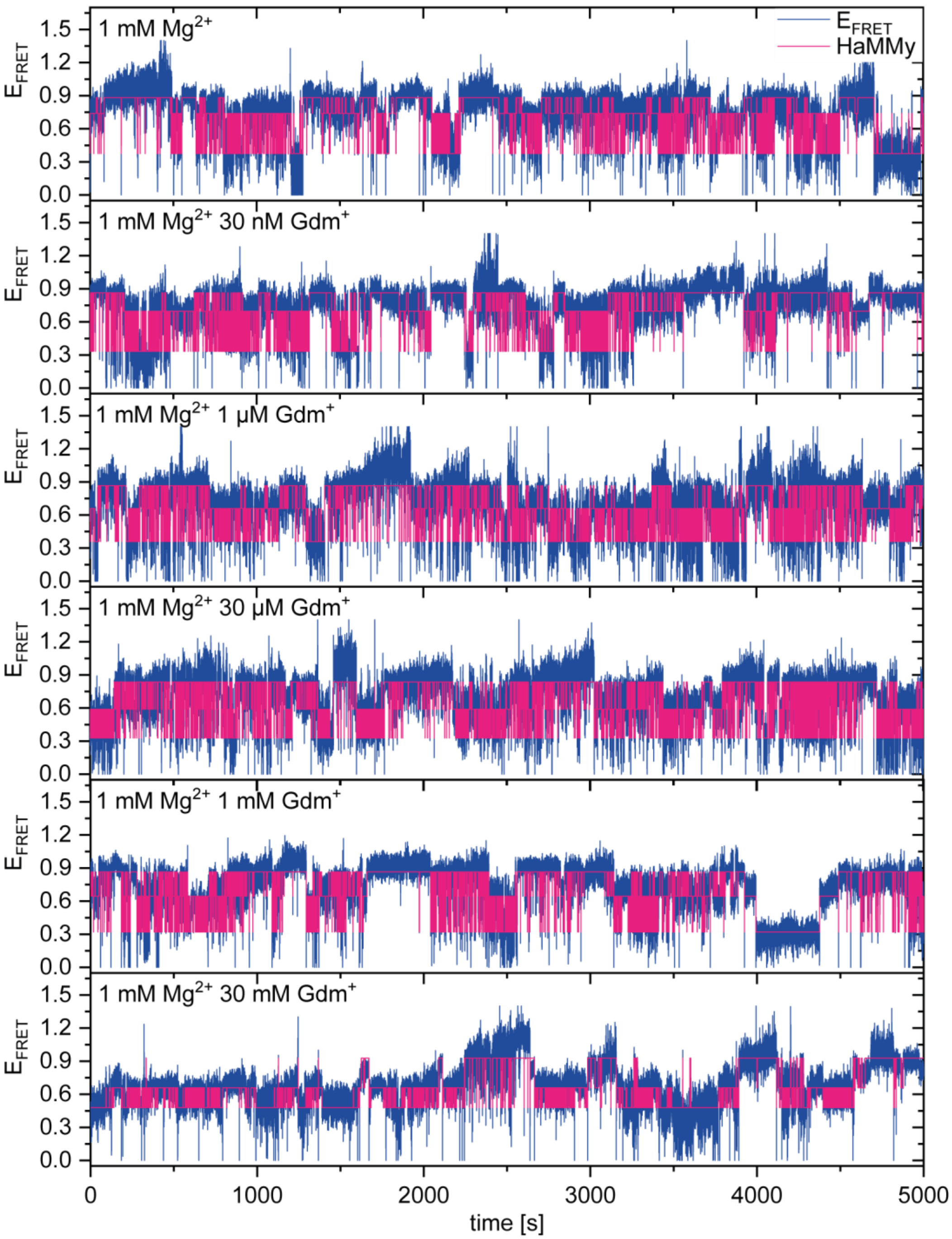

Supplementary Figure S11. Stitched and HaMMy fitted smFRET traces from the Gdm^{+} titration at constant 1 mM Mg^{2+} used for kinetic analysis. FRET trajectories were selected manually and stitched to a single trace with 50000 datapoints (blue). Each trace was subjected to a 3 state Hidden Markov modelling using HaMMy (pink).
